# Infrared and Visible Image Fusion Network Based on Self-Compensating Lightweight Convolution

**DOI:** 10.3390/s26123748

**Published:** 2026-06-12

**Authors:** Ruolin Li, Hongmei Wang, Qiaorong Wu, Cheng Liang, Haoyu Li, Jingyu Wang

**Affiliations:** School of Astronautics, Northwestern Polytechnical University, Xi’an 710072, China; li_ruo_lin@mail.nwpu.edu.cn (R.L.);

**Keywords:** image fusion, model lightweighting, convolutional neural networks, depthwise separable convolution

## Abstract

Deep learning has significantly improved the quality of infrared and visible image fusion. However, existing mainstream deep fusion networks often come with complex architectures and a large number of parameters. While general lightweight techniques can effectively reduce model complexity, they often weaken feature interactions during the lightweighting process, resulting in the loss of complementary texture and thermal information in fused images and making it difficult to balance fusion performance and model efficiency. To address these issues, this paper constructs an infrared and visible image fusion network based on a self-compensating lightweight convolution mechanism, named LWC-DenseFuse. The core of the network lies in a self-compensating lightweight convolution module, which goes beyond conventional convolution replacement and explicitly addresses feature degradation introduced by lightweight design. The module decouples spatial and channel correlations of standard convolution through depthwise convolution and pointwise convolution, while incorporating a channel attention mechanism to adaptively enhance salient features. Additionally, channel shuffle technology is employed to promote information exchange between groups, thereby enhancing feature interaction and compensating for the loss of critical information caused by lightweight design. To further improve the representation capability of the lightweight network during optimization, a staged training strategy with progressive loss weighting is introduced. Experimental evaluations demonstrate that the proposed fusion network significantly reduces the number of model parameters while ensuring real-time inference performance. Meanwhile, it effectively alleviates the performance degradation typically associated with lightweight architectures, as evidenced by improvements in information entropy and visual fidelity.

## 1. Introduction

In recent years, deep learning-based infrared and visible image fusion methods have achieved remarkable results in fields such as target detection, security surveillance, and military reconnaissance, owing to their powerful feature extraction and nonlinear fitting capabilities. Among them, the autoencoder-based fusion framework [1] learns deep features from source images and reconstructs the fused image, outperforming traditional methods in preserving both texture details and thermal radiation information. However, as fusion network performance improves, the complexity of model architectures also increases. Existing mainstream fusion networks typically rely on stacking numerous standard convolutional layers to ensure sufficient feature extraction. While this design enhances fusion accuracy, it also introduces significant parameter redundancy and high computational costs. For applications with stringent real-time requirements or hardware-constrained computing devices, these bulky models often fail to meet practical deployment needs.

To improve the deployment efficiency of deep neural networks, numerous lightweight architectures have been proposed, such as MobileNet [2] and GhostNet [3]. MobileNet reduces computational complexity by decomposing standard convolutions into depthwise and pointwise operations, while GhostNet generates redundant feature representations through inexpensive linear transformations to further improve efficiency. These methods have demonstrated remarkable success in various high-level vision tasks, such as object detection, tracking, and image classification. However, such efficiency-oriented designs typically inevitably weaken feature interactions across channels.

Unlike high-level vision tasks, infrared–visible image fusion requires not only semantic representation but also the preservation of complementary texture, structural, and thermal information from different modalities. Therefore, sufficient cross-channel feature interaction is essential for preserving fine-grained textures and salient thermal targets in fused images. However, directly introducing existing lightweight strategies into image fusion networks may weaken complementary feature aggregation during feature extraction, resulting in texture degradation and blurred target boundaries. Moreover, the limited representation capacity of lightweight fusion networks makes the optimization process more sensitive to loss balancing and training strategies, further aggravating the difficulty of achieving stable reconstruction quality.

To address these issues, we propose a self-compensating lightweight fusion framework, termed Lightweight Convolution DenseFuse (LWC-DenseFuse). Specifically, a self-compensating lightweight convolution (LWC) module is designed to decouple spatial and channel correlations for efficient feature extraction while compensating for information loss introduced by lightweight operations. Based on the DenseFuse architecture, the proposed LWC module is incorporated into both feature extraction and reconstruction stages, enabling an effective balance between computational efficiency and fusion performance. To further alleviate optimization instability under limited model capacity, a staged training strategy with progressive loss weighting is also introduced to enhance convergence behavior and improve reconstruction quality.

The main contributions of this paper are summarized as follows:

1. A self-compensating lightweight convolution (LWC) module is proposed to address the inherent loss of complementary information caused by lightweight convolutional operations. Unlike conventional lightweight designs that focus primarily on computational reduction, the proposed LWC introduces an information compensation mechanism to preserve and enhance discriminative feature interactions during feature extraction, thereby improving the representation capability of lightweight fusion networks.
2. A novel lightweight infrared–visible image fusion framework, termed LWC-DenseFuse, is developed based on the proposed LWC module. By embedding self-compensating feature interaction into both feature extraction and reconstruction stages, the proposed framework establishes an information-compensation-guided lightweight fusion paradigm, enabling substantial model compression while maintaining effective cross-modal information preservation. In addition, a staged training strategy with progressive loss weighting is designed to further enhance optimization stability and fusion performance.
3. Extensive experiments on multiple benchmark datasets demonstrate the effectiveness of the proposed framework. The proposed method achieves a superior balance between fusion quality and computational efficiency, consistently outperforming representative lightweight fusion approaches while requiring significantly fewer parameters and FLOPs. Moreover, ablation studies verify the effectiveness of the proposed training strategy in improving reconstruction quality and visual fidelity.

## 2. Related Work

### 2.1. Infrared and Visible Image Fusion

In recent years, as deep learning technology has demonstrated high accuracy and strong adaptability across various domains, deep neural network models have gradually been applied to the field of infrared and visible image fusion. Deep learning-based infrared and visible image fusion has mainly evolved into several categories: methods based on convolutional neural networks [4,5], methods based on autoencoders [1,6], methods based on generative adversarial networks [7,8], and methods based on Transformers [9,10]. Among these, methods based on autoencoders have gained widespread application.

The early DeepFuse model [1] demonstrated the feasibility of using CNNs as the encoder part of an autoencoder, yet it suffered from the loss of deep-level information. To address this bottleneck, researchers worldwide have conducted a series of fruitful explorations: Huang [11] pioneered the introduction of densely connected blocks into the network, enhancing gradient and information flow and alleviating information loss in deep networks. The SeAFusion framework proposed by Tang [12] employs joint adaptive cyclic training of the fusion network and a segmentation network, enabling the fusion results to directly serve downstream detection tasks and significantly improving the model’s scene understanding capability. Li [13] further utilized a saliency segmentation network to generate target masks, separating foreground and background and effectively suppressing interference from redundant information. Additionally, Zhao [14] introduced self-supervision and feature adaptation mechanisms into the network, while reference [15] adopted a deep interpretable dual-encoder structure and a cyclic cotraining strategy, optimizing the feature extraction and reconstruction processes from different perspectives. Although autoencoder-based methods have made significant progress in performance, the inherent challenges of their encoder–decoder structure—such as high training complexity and strict requirements for data alignment—remain major obstacles in practice.

### 2.2. Current Status of Model Lightweighting for Image Fusion

With the continuous development in the field of infrared and visible image fusion, the depth and complexity of fusion models have also been increasing. The enormous number of parameters and computational requirements have become major bottlenecks limiting their further advancement. Therefore, introducing model lightweighting techniques into the image fusion domain to compress models and improve efficiency while maintaining fusion performance has become an important research direction.

#### 2.2.1. Current Status of Model Lightweighting Research

Currently, mainstream neural network model compression methods include: Network Pruning, Parameter Sharing, Low-Rank Decomposition, Knowledge Distillation, and Compact Network Design. As hardware capabilities evolve and system complexity increases, to meet the demands of model deployment, compact architecture design has become one of the most rapidly developing categories within model compression methods.

Iandola [16] proposed in 2016 to replace the traditional convolutional structure with a Fire Module containing Squeeze and Expand layers, and based on this, designed SqueezeNet. The Squeeze layer contains only 1 × 1 convolutions to reduce parameters and channels, while the Expand layer includes both 1 × 1 and 3 × 3 convolutional kernels. Google’s MobileNet V1 [2] employed depthwise separable convolutions. In this model, depthwise convolution applies a single convolution kernel per input channel for feature extraction, while pointwise convolution uses 1 × 1 convolutions to fuse features from different channels and alter the channel count. MobileNet V1 significantly reduced the network’s parameter count and computational load and was successfully applied to tasks such as face recognition. To further enhance network performance, Google subsequently introduced the MobileNet V2 [17] and MobileNet V3 [18] models, progressively incorporating residual structures and attention modules to improve operational speed. Huawei Noah’s Ark Lab, based on the similarity of image feature maps, proposed the Ghost module [3]. In this module, a series of linear transformations are applied to a small number of original feature maps to generate multiple “ghost” feature maps, thereby obtaining rich features with minimal computation. GhostNet, built by stacking Ghost modules, achieved higher classification accuracy than MobileNet V3 under comparable computational loads. Subsequently, Noah’s Ark Lab introduced GhostNet V2 [19] and GhostNet V3 [20] models, further enhancing network operational efficiency.

Lightweight convolutional network design methods compress deep neural networks at the convolutional structure level and can achieve excellent compression effects for specific tasks. However, the generalization capability of compressed models is relatively weak, and their expressive power tends to decrease compared to the original models.

#### 2.2.2. Current Status of Lightweighting Methods in the Field of Image Fusion

With the rapid development of infrared–visible image fusion and lightweight network design, increasing attention has been devoted to reducing model complexity while preserving fusion performance. Early lightweight fusion methods mainly focused on replacing standard convolutions with efficient operators. For example, Cheng et al. [21] proposed UNIFusion, which integrates the Ghost module into an autoencoder architecture and employs mutual guided filtering to decompose source images into base and detail layers, thereby reducing computational cost while retaining detailed information. Similarly, depthwise separable convolutions have been introduced to decouple spatial and channel-wise feature extraction, significantly decreasing parameter redundancy while maintaining reconstruction capability [22].

More recently, lightweight fusion has evolved beyond convolutional optimization toward compact representation learning. LUT-Fuse [23] distills a multimodal fusion network into a learnable lookup table, encoding low-level features and high-level contextual priors into discrete mappings to achieve non-iterative and highly efficient inference. Meanwhile, several dedicated lightweight fusion frameworks, including SFDFuse [24], HaarFuse [25], further reduce computational complexity through dual-domain feature modeling or wavelet-based representations while maintaining competitive fusion performance.

However, despite their effectiveness, these approaches still exhibit two major limitations:

(1) Structural decoupling in Ghost- and depthwise-based methods inevitably weakens cross-channel and cross-modal feature interactions, which are essential for preserving complementary thermal and texture information. This limitation also makes feature optimization more sensitive to training dynamics under lightweight constraints.
(2) LUT-based methods rely on highly compact discrete representations, which reduce modeling flexibility and hinder the preservation of fine-grained structural details during fusion, thereby increasing the difficulty of stable reconstruction during training.
(3) Frequency-domain-based methods rely on explicit spectral transforms, which constrain joint spatial–channel representation learning and weaken cross-modal interaction modeling, leading to suboptimal convergence behavior in end-to-end optimization.

To address these issues, a lightweight infrared–visible image fusion framework, termed LWC-DenseFuse, is proposed based on a self-compensating lightweight convolution (LWC) module. By incorporating a self-compensating feature interaction mechanism into both feature extraction and reconstruction stages, the proposed framework mitigates the degradation of cross-channel and cross-modal information exchange caused by lightweight operations, enabling substantial model compression while preserving complementary texture and thermal information. To further improve optimization stability under these limitations, a staged training strategy with progressive loss weighting is introduced to enhance convergence and reconstruction quality under limited model capacity.

## 3. Materials and Methods

### 3.1. Overall Network Architecture

**Figure 1:**
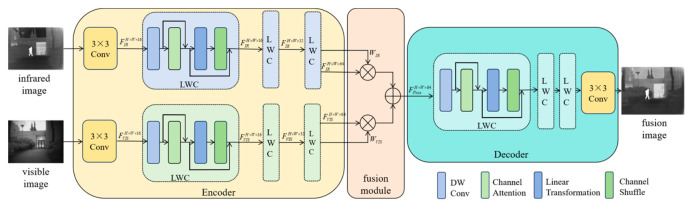
Schematic Diagram of the LWC-DenseFuse Network Architecture.

To construct a lightweight network specifically designed for infrared and visible image fusion, this paper conducts an in-depth redesign based on the classic DenseFuse encoder–decoder framework and proposes a lightweight infrared and visible image fusion network—LWC-DenseFuse. The overall architecture of the network is illustrated in Figure 1.

As shown in Figure 1, the proposed LWC-DenseFuse network consists of three components: an encoder, a fusion layer, and a decoder. Given an infrared image and a visible image as inputs, shallow feature extraction is first performed using a 3 × 3 convolution to generate 16 channel feature representations, denoted as $F_{IR}^{H\times W\times 16}$ and $F_{VIS}^{H\times W\times 16}$. The resulting shallow features are then sequentially processed by three cascaded LWC modules. The output channel numbers of the three LWC modules are 16, 32, and 64, respectively, enabling the network to capture semantic information at different receptive field scales. Through this progressive feature extraction process, the deep infrared feature map $F_{IR}^{H\times W\times 64}$ and visible feature map $F_{VIS}^{H\times W\times 64}$ are obtained. In the fusion stage, L1-norm-based activity measures are computed from $F_{IR}^{H\times W\times 64}$ and $F_{VIS}^{H\times W\times 64}$ to generate corresponding weight maps $W_{IR}$ and $W_{VIS}$, which are subsequently used to perform weighted feature fusion and produce the fused feature representation $F_{Fuse}^{H\times W\times 64}$. Finally, $F_{Fuse}^{H\times W\times 64}$ is fed into the decoder, which consists of three cascaded LWC modules and a final convolution layer, to progressively reconstruct the spatial details and generate the fused image.

### 3.2. Lightweight Convolution Module Design

**Figure 2:**
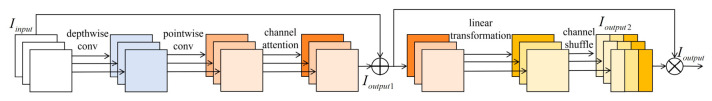
Schematic Diagram of the Lightweight Convolution Module.

The lightweight convolution module proposed in this paper is centered around depthwise convolution and pointwise convolution, incorporating operations such as linear transformation, channel shuffle, and residual connections. The module takes a 16 channel feature map as input. The depthwise convolution adopts a 3 × 3 kernel with stride 1 and padding 1, followed by a 1 × 1 pointwise convolution. A 1 × 1 linear transformation is then applied to adjust channel dimensions, while channel shuffle with a group size of 2 enhances inter-channel information interaction. This design significantly reduces model complexity while preserving feature representation capability. The overall structure of the module is illustrated in Figure 2.

The lightweight convolution module proposed in this paper follows the principle of “compress first, then compensate,” and is divided into two parts: lightweight design and information compensation. The lightweight design component, centered on depthwise convolution and pointwise convolution combined with linear transformation, achieves efficient compression of the model. The information compensation part utilizes operations such as channel attention and channel shuffle to offset information loss, ensuring feature representation capability while significantly reducing model complexity. The structure of this module is illustrated in Figure 2.

In the lightweight design, the use of depthwise convolution, pointwise convolution, and linear transformation significantly reduces the model’s parameter count and computational load, which is key to achieving model lightweighting. To further adapt to the specific task of infrared and visible image fusion, compensate for the channel information fragmentation caused by depthwise separable convolution and linear transformation operations, and enhance the network’s ability to filter key features, this paper designs an information compensation module while compressing the model structure. This includes introducing a channel attention mechanism, channel shuffle, and residual connections. Among these, the channel attention mechanism dynamically weights each channel based on the importance of its information, highlighting critical features while suppressing less important ones. The channel shuffle effectively enhances information flow and combination among feature channels, improving feature diversity and discriminability. The introduction of residual connections helps preserve the original information from the input image to a certain extent, mitigating the information loss caused by feature compression.

The information flow in this process can be summarized by the following general formula:

(1) $$I_{output}=Concat(I_{output1},I_{output2})$$

$I_{output}\in {\mathbb{R}}^{N\times H\prime \times W\prime }$ represents the output image, $I_{output1}\in {\mathbb{R}}^{N/2\times H\prime \times W\prime }$ be the output image after depth-aware module and $I_{output2}\in {\mathbb{R}}^{N/2\times H\prime \times W\prime }$ be the image after the channel interaction module.

(2) $$I_{output1}=SE((I_{input}*f_{1})⊙f_{2})+(I_{input}⊙f_{4})$$

(3) $$I_{output2}=Shuffle_{g}(I_{output1}*f_{3})$$

$I_{input}\in {\mathbb{R}}^{M\times H\times W}$ be the input image, ∗ denotes the depthwise convolution process, and ⊙ denotes the pointwise convolution process. SE(·) refers to the processed output after passing through the module, and $Shuffle_{g}(\cdot )$ represents the channel shuffle operation with a group size of g. The kernels used in the three convolutions are $f_{1}$, $f_{2}$, $f_{3}$ and $f_{4}$, where the sizes of $f_{1}$ and $f_{3}$ are K×K, and the size of $f_{2}$ and $f_{4}$ is 1×1.

The LWC module primarily consists of depthwise convolution and pointwise convolution, with an overall compression ratio of:

(4) $$r=\frac{p_{0}}{p_{1}+p_{2}+p_{3}+p_{4}}$$

The final parameter compression ratio is denoted by r, where $p_{0}$ represents the parameter count of standard convolution with M input channels and N output channels, $p_{1}$ the parameter count of depthwise convolution with M input channels, $p_{2}$ the parameter count of half-channel pointwise convolution with M input channels and N/2 output channels, $p_{3}$ the parameter count of spatial linear transformation with N/2 input channels, and $p_{4}$ denotes the parameter count of the SE attention module, which consists of two fully connected layers with a reduction ratio of *s*. Ignoring bias terms, the number of parameters equals the product of input dimension and output dimension.

In the above convolution process, assuming the input image size is M×H×W, the output image size is N×H′×W′, and the kernel sizes in the depthwise convolution, pointwise convolution, and linear transformation processes are K×K×M, $M\times \frac{N}{2}$ and $K\times K\times \frac{N}{2}$, respectively. The kernel size for standard convolution is K×K×N, and the compression factor for the channel attention calculation is 8. The values for $p_{0}$, $p_{1}$, $p_{2}$, $p_{3}$ and $p_{4}$ are calculated as follows:

(5) $$p_{0}=N\cdot K\cdot K\cdot M$$

(6) $$p_{1}=K\cdot K\cdot M$$

(7) $$p_{2}=M\cdot \frac{N}{2}$$

(8) $$p_{3}=K\cdot K\cdot \frac{N}{2}$$

(9) $$p_{4}=\frac{\left(N/2\right)}{s}\cdot \frac{N}{2}\cdot 2=\frac{N^{2}}{2s}$$

where *M* denotes the number of input channels, *N* denotes the number of output channels, *K* denotes the convolution kernel size, and *s* denotes the scaling factor of the auxiliary operation. In this work, we take the case *M* = *N* = 16 as an illustrative example. For other LWC modules with different channel configurations, the compression ratio can be calculated similarly.

The final compression ratio of the lightweight convolution module is:

(10) $$r=\frac{K\cdot K\cdot M+M\cdot \frac{N}{2}+K\cdot K\cdot \frac{N}{2}+\frac{N^{2}}{2s}}{N\cdot K\cdot K\cdot M}=\frac{1}{N}+\frac{1}{2K^{2}}+\frac{1}{2M}+\frac{N}{2sK^{2}M}$$

**Figure 3:**
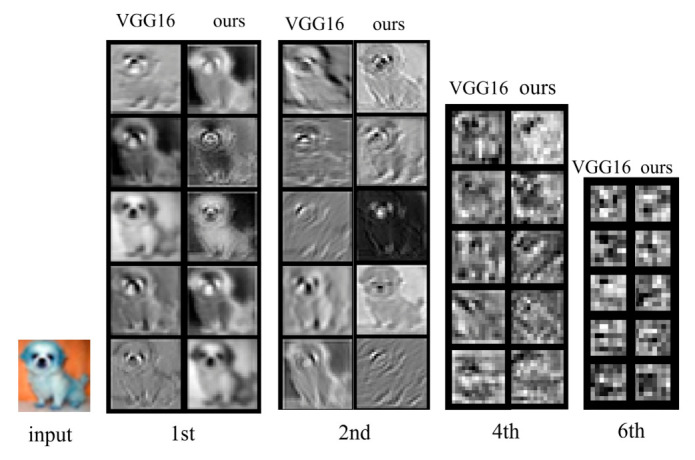
Comparison of Feature Maps Before and After Applying Lightweight Convolution.

To verify the feature extraction capability of the proposed lightweight convolution module, it is applied to the VGG-16 network for comparison with standard convolution. A comparison of feature maps output by the 1st, 2nd, 4th, and 6th convolutional layers of the VGG-16 network before and after compression is shown in Figure 3.

The visualization results in Figure 3 demonstrate that the designed lightweight convolution kernel not only has fewer parameters than the standard convolution in the baseline model but also still outputs feature maps similar to those of standard convolution. This serves as an essential prerequisite for correctly extracting target features and accomplishing infrared and visible image fusion.

In summary, the proposed lightweight convolution module is based on the principle of first decoupling and compressing, then interacting and compensating. Depthwise separable convolution and linear transformation achieve efficient feature compression, while channel attention and channel shuffle operations effectively compensate for the loss in feature representation capability of lightweight convolution from the perspectives of feature recalibration and inter-group information interaction, respectively.

### 3.3. Loss Function

To ensure that the encoder and decoder can effectively extract features and reconstruct high-quality images, this paper adopts an unsupervised autoencoder training paradigm. During training, not only is a composite loss function designed, but a novel staged training strategy is also introduced to optimize the network’s convergence speed and reconstruction quality.

The total loss function of the proposed network is formulated as:

(11) $$L_{total}=L_{pixel}+\lambda L_{ssim}$$

where $L_{pixel}$ represents the pixel-level loss, $L_{ssim}$ denotes the structural similarity (SSIM) loss, and λ is a parameter used to balance the two types of loss.

The pixel loss employs the mean squared error (MSE) to constrain the pixel intensity consistency between the input image $I_{in}$ and the reconstructed image $I_{out}$, ensuring that the reconstructed image closely approximates the source image in terms of overall brightness and contrast. The formula is as follows:

(12) $$L_{pixel}={\left\|I_{in}-I_{out}\right\|}_{2}^{2}$$

To overcome the drawback of MSE, which tends to cause edge blurring in images, the SSIM loss is introduced to enhance the network’s ability to preserve texture details and edge information. Its calculation formula is:

(13) $$L_{ssim}=1-SSIM(I_{in},I_{out})$$

where SSIM(.) [26] denotes the structural similarity index measure. The SSIM loss is employed to preserve luminance, contrast, and structural information, encouraging the re-constructed image to maintain high perceptual fidelity to the input image. $I_{in},I_{out}$ has the same meaning as above.

During training, to address the relatively limited fitting capacity of lightweight networks and to ensure fusion image quality, a staged parameter λ is adopted, with the following specific values:

(14) $$\lambda =\left\{\begin{array}{cc} 1 & \;\;epoch\leq 100\\ 10 & \;\;100<epoch\leq 150\\ 100 & \;\;epoch>150 \end{array}\right.$$

The training process is divided into three stages based on the epoch count. The first stage aims to guide the network in rapidly learning the basic features and brightness distribution of the image, avoiding unstable gradients in the early training phase due to complex structural constraints. In the second stage, the weight of the structural similarity loss is increased for further optimization, fine-tuning features, sharpening image edges, and restoring high-frequency texture details. In the final stage, the balance between the two losses is further intensified to enhance image reconstruction quality.

## 4. Experiment

### 4.1. Experimental Setup

All experiments are implemented in PyTorch 1.12 with Python 3.9 on a Windows 10 system equipped with an NVIDIA GeForce RTX 3060 Ti GPU. The model is trained using the Adam optimizer with an initial learning rate of $1\times 10^{-4}$, a batch size of 16, and a weight decay of $1\times 10^{-5}$, for a total of 200 epochs; the learning rate is halved every 50 epochs. The MS-COCO dataset is used for training, while the TNO, MSRS, and LLVIP datasets are adopted for testing. All input images are resized to 256×256. For the MSRS and LLVIP datasets, RGB visible images are converted to grayscale to match single-channel infrared images, with only luminance information retained for fusion.

Following the standard unsupervised autoencoder training strategy used in DenseFuse [1] and its variants, we train only the encoder and decoder on the MS-COCO dataset [27] without using any infrared or visible paired images. Specifically, all MS-COCO images (originally RGB) are converted to single-channel grayscale. Each grayscale image is resized to 256 × 256 and used as both the input and reconstruction target of the autoencoder, which is trained to learn single-channel image reconstruction without involving any infrared data. This pre-trained autoencoder is later shared across all fusion tasks, where the fusion layer is inserted between the encoder and decoder.

For evaluation, we use three publicly available infrared and visible image fusion datasets: TNO, MSRS, and LLVIP. Specifically, the TNO dataset contains a total of 60 image pairs, from which we selected 41 pairs for testing. The MSRS dataset comprises 361 test image pairs, each having a resolution of 640 × 480, all of which were utilized in our experiments. The LLVIP dataset contains a total of 15,488 aligned infrared and visible image pairs, each having a resolution of 1280 × 720, and we used 11 pairs for quantitative evaluation. For the MSRS and LLVIP datasets, RGB visible images are converted to grayscale using the same method as the training phase to match the single-channel infrared images, retaining only luminance information for fusion.

Quantitative evaluation is conducted using average gradient (AG), information entropy (EN), visual information fidelity (VIF), edge strength, mutual information (MI), parameter count (Params/M), floating-point operations (FLOPs/G), and average inference time (Avg_time/s).

### 4.2. Ablation Study

#### 4.2.1. Module Ablation

This subsection conducts an ablation study based on the lightweight design component of the proposed LWC-DenseFuse model, focusing on the channel attention mechanism and the channel shuffle operation in the information compensation strategy. Both subjective and objective evaluations are performed on the fusion results to validate the importance of the information compensation strategy in infrared and visible image fusion networks, as well as the effectiveness of each component designed in this paper. The following model notations are used:

M1—Lightweight design only;

M2—Lightweight design + Channel Attention;

M3—Lightweight design + Channel shuffle;

M4—The complete LWC-DenseFuse proposed in this paper.

**Figure 4:**
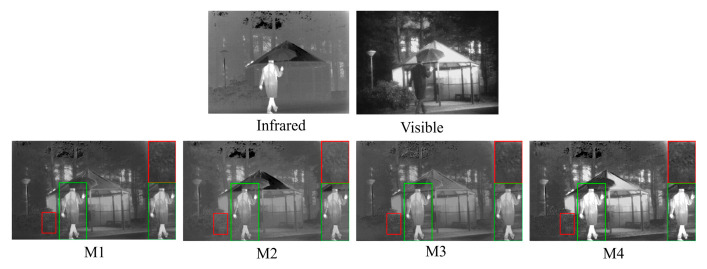
Results of the Ablation Study. The red box denotes fine-grained image details, while the green box marks salient target regions.

This ablation study was conducted on the TNO dataset, which consists of grayscale images. Figure 4 shows a schematic diagram of the fusion results for each model.

As can be seen from Figure 4, although the baseline model M1 is able to fuse the basic information from infrared and visible images, the fusion result appears generally dark, with insufficient prominence of infrared targets and blurry or lost texture details in background elements such as leaves and branches. After introducing the channel attention mechanism, model M2 shows a certain enhancement in the saliency of bright infrared targets and some suppression of background noise, yet there remains room for improvement in preserving texture details. Model M3, which incorporates the channel shuffle operation, promotes feature interaction across different channels, resulting in a slight improvement in the textural richness of the fused image. However, due to the lack of guidance from the attention mechanism, the overall brightness distribution appears somewhat uneven in certain areas.

In contrast, the full model M4 proposed in this paper demonstrates the best visual performance. Benefiting from the synergistic effect of the attention mechanism and channel shuffle, the fused image generated by M4 not only clearly preserves the thermal radiation targets from the infrared image with sharp edges and well-defined contours but also seamlessly integrates the rich background textures and detailed information from the visible image.

**Figure 5:**
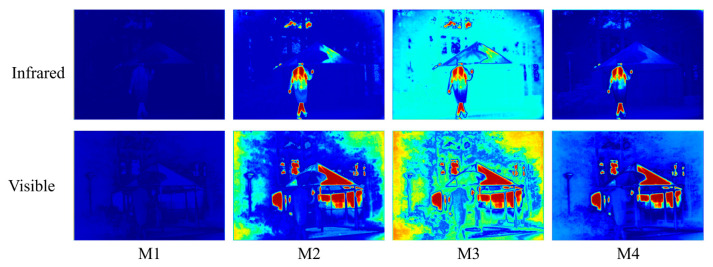
Heatmaps of Deep Encoder Features for Each Model. Red regions correspond to highly activated and concentrated feature attention, while blue regions indicate weakly activated and less-attended features.

To more intuitively validate the above conclusions and investigate the impact of different information compensation strategies on feature extraction, this section presents a visualized heatmap analysis of the deep-layer feature maps output by the encoders of the four models, as shown in Figure 5.

In model M1, which relies solely on lightweight design, the extracted feature responses are extremely weak. Whether in the infrared or visible channel, both target contours and background textures are nearly invisible, demonstrating that the interruption of inter-channel information exchange leads to severe loss of critical information during transmission. After introducing the channel attention mechanism, the human target area in the infrared channel begins to receive attention, but the texture response of visible features remains relatively weak. This indicates that while the attention mechanism can effectively enhance the saliency of high-contrast targets, it struggles to focus on complex spatial details. With the introduction of the channel shuffle operation, the overall brightness of visible features increases, and textural details such as trees and pavilions begin to emerge. This shows that channel shuffle breaks down information barriers between components, but the salient human target becomes overshadowed by background noise, lacking specificity.

In the complete model M4 proposed in this paper, the infrared channel shows a bright dark red highlighting the human target, with background noise effectively suppressed, demonstrating strong target capture capability. In the visible channel, textures such as the outline of the pavilion are captured and appear sharper and cleaner compared to M3. This proves that the synergistic effect of the two detail compensation strategies enables the lightweight network to extract highly discriminative and robust complementary features.

The specific metrics of the ablation study are presented in Table 1.

Analysis of the results in Table 1 demonstrates that the proposed M4 model consistently achieves superior performance across most key evaluation metrics, thereby substantiating the effectiveness and contribution of each component within the LWC module.

The proposed LWC-DenseFuse model achieves optimal performance in terms of EN, AG, VIF, and Qabf. This indicates that the introduced channel attention mechanism successfully focuses on high-frequency textures in the source images, while the channel shuffle operation effectively promotes feature fusion. As a result, the final fused image contains the richest information, the clearest texture details, and the closest approximation to an ideal fusion effect in terms of human visual perception. The MI value shows a slight decrease compared to the baseline model and the method using only channel attention. This is because mutual information primarily measures the statistical correlation in pixel distribution between the fused image and the source images. Model M1, which employs only the lightweight design module, significantly reduces the number of parameters. However, its inherent limitation of cutting off direct inter-channel interaction leads to noticeable information loss, making it difficult to achieve high-quality image fusion independently. In contrast, M4 adopts the lightweight design while incorporating information compensation strategies. Although these feature reconstruction and enhancement operations improve visual contrast, they slightly reduce the statistical pixel-level dependency on the source images. Compared to M1, M2, and M3, the proposed M4 inevitably experiences a parameter increase due to the introduction of additional modules, but this increase remains within a controllable range. The inference time of M4 is slightly higher than that of the other methods. Although the number of parameters is reduced, the attention mechanism and channel shuffle introduce additional memory access and computational steps, leading to a slight increase in physical inference time. Nevertheless, the inference time remains at the millisecond level, meeting real-time requirements.

In summary, the channel attention mechanism and channel shuffle strategy effectively compensate for the information loss caused by the lightweight design process, thereby improving image fusion quality. This further proves that the strategy of “compress first, then compensate” is effective, and that the proposed internally compensated lightweight convolution module and the LWC-DenseFuse network constitute a reliable choice that balances both lightweight design and high performance.

#### 4.2.2. Training Strategy Ablation

To validate the effectiveness of the proposed staged SSIM weight scheduling strategy, ablation experiments are conducted by comparing five different weight assignment schemes. All models are trained on 2000 images randomly sampled from a collection of 8279 natural images, converted to grayscale and resized to 256 × 256 pixels. Training is performed for 60 epochs using the Adam optimizer with a learning rate of 1 × 10^−5^ and a batch size of 4.

The evaluated strategies include:

(A) Staged, the proposed progressive scheme, where the SSIM weight λ is set to 0 during epochs 0–19, increased to 10 during epochs 20–29, and further raised to 100 during epochs 30–59, following a reconstruction-first and structure-aware optimization paradigm;
(B) Constant-Low, which maintains λ = 10 throughout training;
(C) Constant-High, which fixes λ = 100 for all epochs;
(D) Linear Annealing, where λ increases linearly from 0 to 100;
(E) Cosine Annealing, where λ follows a cosine progression from 0 to 100. All strategies employ the same DenseFuse architecture enhanced with GhostModules, ensuring that performance differences originate solely from the weight scheduling mechanism.

For evaluation, each trained model is tested on four publicly available infrared–visible image fusion benchmarks, including LLVIP, MSRS, Roadscene, and TNO. Five complementary metrics (PSNR, MI, VIF, SCD, and Qabf) are adopted to assess pixel-level fidelity, information preservation, perceptual quality, structural consistency, and overall fusion performance, respectively.

**Figure 6:**
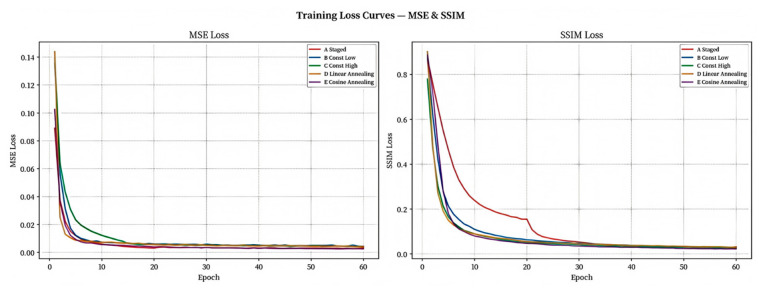
Strategic Ablation Loss.

The training loss curves under different scheduling strategies are illustrated in Figure 6. For the MSE loss, all strategies exhibit stable convergence behavior, while the proposed staged strategy converges more rapidly in the later training stage due to the gradual emphasis shift from pixel reconstruction to structural optimization. Regarding the SSIM loss, although the proposed strategy introduces abrupt weight transitions at predefined stages, no noticeable training instability or loss oscillation is observed. The Constant-Low strategy demonstrates stable optimization but relatively slow structural convergence. In contrast, the Constant-High strategy accelerates SSIM optimization but may adversely affect early-stage training stability. Both Linear Annealing and Cosine Annealing provide smoother transitions between optimization objectives, achieving a reasonable compromise between stability and convergence speed.

**Figure 7:**
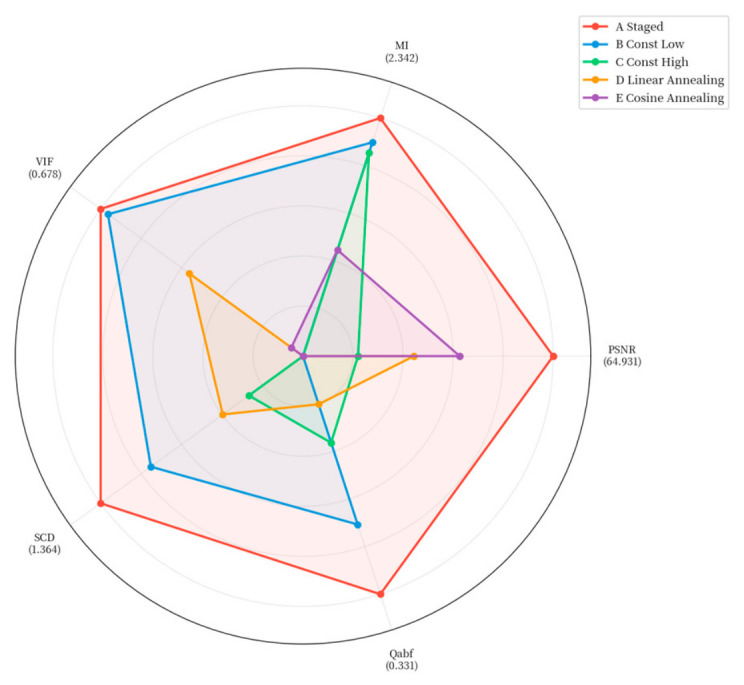
Objective Indicators of Each Strategy on Four Datasets.

The quantitative results shown in Figure 7 further demonstrate the superiority of the proposed staged scheduling strategy. Among all compared schemes, Strategy A consistently achieves the best overall performance across the five evaluation metrics, surpassing constant-weight and continuous annealing alternatives. These results indicate that progressively introducing structural constraints after sufficient reconstruction learning enables the network to better balance detail preservation and structural representation, thereby yielding superior infrared–visible fusion performance.

### 4.3. Comparative Experiment on Different Lightweight Convolution Strategies

**Figure 8:**
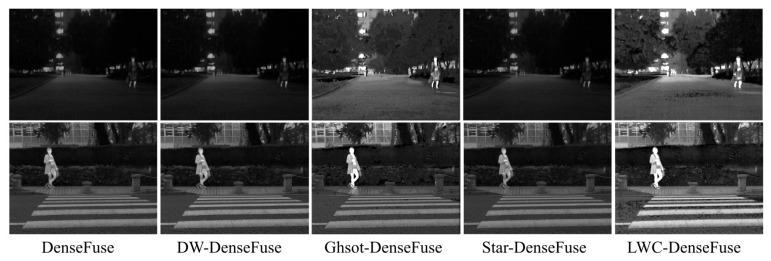
Comparison of Lightweight Convolution Methods on the MSRS Dataset.

**Figure 9:**
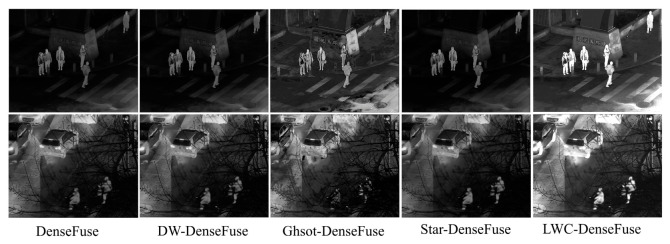
Comparison of Lightweight Convolution Methods on the LLVIP Dataset.

To evaluate the effectiveness of the proposed lightweight design, this subsection compares the proposed lightweight convolution module with existing lightweight methods. The networks included in the comparative experiments are: the original DenseFuse network, the DW-DenseFuse network based on depthwise separable convolution, the Ghost-DenseFuse network based on the Ghost module, the Star-DenseFuse network based on Star convolution [24], and the proposed LWC-DenseFuse network. Comparative experiments are conducted on the MSRS and LLVIP datasets, and the results are presented in Figure 8 and Figure 9, respectively.

Figure 8 presents a comparative analysis of fusion results obtained by different lightweight methods on the MSRS dataset. Although the original DenseFuse network achieves image fusion, the overall image quality is suboptimal, with blurred contours of trees and buildings in the background and insufficient prominence of infrared targets. The DW-DenseFuse network, which incorporates depthwise separable convolution, and the Star-DenseFuse network, which employs the Star method, produce results similar to those of the original DenseFuse, with slight improvements in thermal radiation information around the face and hands of the human target. However, the contrast remains low, the fused images appear overall dark, and detailed information is difficult to distinguish. In comparison, the Ghost-DenseFuse network, which integrates the Ghost module, shows a notable improvement in contrast over the other three methods. Nevertheless, the generated images exhibit overly smoothed textures, reduced edge sharpness, and background details that are even less clear than those produced by the original DenseFuse network.

In contrast, the proposed LWC-DenseFuse method demonstrates the best performance in this scenario. Infrared thermal radiation targets such as pedestrians and vehicles are highlighted with sharp, bright edges, significantly enhancing the overall contrast and brightness of the image. Simultaneously, it delicately restores environmental textures in the nighttime background, achieving an optimal balance between target saliency and background clarity.

Figure 9 presents the fusion results of various lightweight methods on the LLVIP dataset, where infrared thermal radiation targets are more pronounced. By comparing the experimental results from each group, it can be observed that the DenseFuse network, DW-DenseFuse network, and Star-DenseFuse network successfully generate fused images, but the images exhibit low contrast, an overall dark tone, and unclear contours of key targets such as buildings, pedestrians, and vehicles along the roadside. Although the Ghost-DenseFuse network generally produces better image quality compared to the aforementioned three networks and retains the morphological features of targets to some extent, it still suffers from issues such as unclear background information, blurring, and color blocks in elements like crosswalks, building walls, and tree branches.

In contrast, the proposed LWC-DenseFuse method demonstrates strong robustness when handling such scenarios. By promoting thorough feature fusion through the channel shuffle strategy, LWC-DenseFuse not only perfectly preserves the saliency of high-brightness targets from the infrared image—such as people and cars, with sharp and clear edges—but also accurately restores background details from the visible image, such as pavement textures and text. Moreover, the generated images exhibit overall higher brightness and clear contrast between targets and the background. This indicates that the designed LWC module can effectively combine the high-brightness characteristics of infrared images with the detailed information from visible images, significantly improving the quality of the fused images.

Table 2 presents a comprehensive performance comparison of various lightweight methods on the MSRS dataset.

Table 2 provides a detailed list of objective evaluation metrics for each lightweight model on the MSRS dataset. Based on the analysis of the table, it is evident that the proposed LWC-DenseFuse method achieves a significant performance leap in both visual quality and information richness.

Specifically, the LWC-DenseFuse method achieves optimal results in three key metrics: average gradient, information entropy, and visual information fidelity. Among these, the advantage in the VIF metric is particularly notable, indicating that after incorporating the LWC module, the fused image achieves the highest fidelity in terms of human visual perception, effectively avoiding image distortion. In contrast, the DW-DenseFuse network, which introduces depthwise separable convolution, shows relatively high values in edge retention and mutual information, but its VIF and AG metrics are comparatively lower. This suggests that DW-DenseFuse tends to mechanically preserve the original pixel distribution of the source images while losing some texture details that align with human visual perception during the feature extraction process. In terms of parameter count and inference time, although the proposed method does not achieve the best results, it attains a highly competitive second-best performance in parameter count. Meanwhile, its inference time differs only marginally from that of the best-performing DenseFuse method, indicating that it still maintains a certain advantage.

Table 3 presents the overall performance comparison of various lightweight methods on the LLVIP dataset.

Table 3 presents the test results of various models on the LLVIP dataset, where infrared targets are more prominent. The data indicate that the advantages of the proposed method are further amplified when handling high dynamic range scenes.

Unlike the MSRS dataset, in the LLVIP dataset, the proposed method not only maintains its leading position in EN and VIF but also surpasses other methods in the edge retention metric, achieving the highest score. This demonstrates that the channel attention mechanism within the LWC module can accurately capture the bright thermal targets in infrared images and transfer their sharp edge information completely to the fused image, thereby overcoming the edge-blurring issue observed in other lightweight methods under strong thermal source scenarios. Compared to DenseFuse, the proposed fusion network LWC-DenseFuse significantly reduces the number of parameters, confirming its effectiveness in eliminating parameter redundancy. Although the inference time increases slightly, it remains within the millisecond range, fully meeting real-time requirements.

In summary, the quantitative results in Table 2 and Table 3 demonstrate that the proposed LWC module achieves a consistently low parameter complexity while preserving high-quality fusion performance. In contrast to conventional lightweight compression schemes that often incur performance degradation, the proposed method yields superior results in key metrics such as AG, EN, and VIF, indicating enhanced preservation of structural and textural information. Although the introduced self-compensation mechanism does not lead to additional improvements in inference speed, the overall runtime remains sufficiently efficient to meet real-time processing requirements. Importantly, the proposed approach offers a substantial reduction in model parameters, providing clear advantages in terms of memory efficiency and deployment feasibility while maintaining competitive computational latency.

### 4.4. Comparative Experiment with Advanced Lightweight Methods

To validate the effectiveness and advancement of LWC-DenseFuse, several representative lightweight infrared–visible image fusion methods are selected for comparison. Specifically, LUT-Fuse, RCAFusion [28], SAGE [29], and UNIFusion are all lightweight fusion frameworks that aim to balance fusion quality and computational efficiency. While LUT-Fuse focuses on deployment-oriented acceleration through learnable lookup tables, RCAFusion emphasizes efficient cross-modal feature interaction, SAGE represents recent lightweight unified fusion architectures, and UNIFusion serves as a representative end-to-end fusion framework. These methods collectively cover the major research directions in contemporary lightweight image fusion. Therefore, comparisons with these approaches provide a comprehensive evaluation of LWC-DenseFuse in terms of fusion performance, computational complexity, and deployment efficiency.

**Figure 10:**
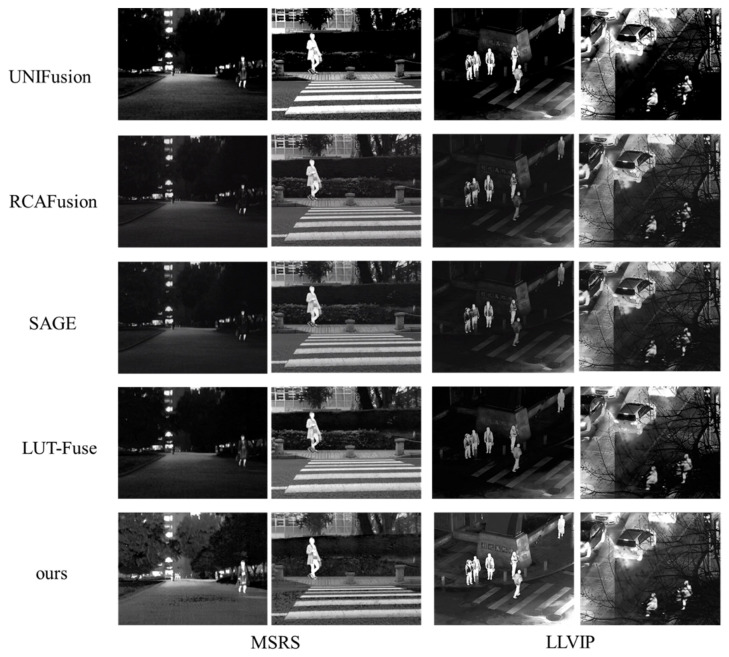
Comparison Results with Different Methods.

Experiments were conducted on the MSRS and LLVIP datasets, as shown in Figure 10. To provide a fair and realistic evaluation of computational complexity, the FLOPs of all compared methods were calculated using the original image resolutions of the respective datasets.

Figure 10 illustrates the qualitative fusion results of different methods on the MSRS and LLVIP datasets. While all compared methods can highlight infrared pedestrian targets, noticeable differences are observed in target saliency enhancement and background detail preservation. LUT-Fuse tends to generate relatively dim fused images, causing the attenuation of fine structures. RCAFusion and SAGE further suffer from excessive brightness suppression, which weakens vegetation textures and pedestrian boundaries. UNIFusion alleviates the brightness degradation issue to some extent, yet background textures and object contours remain over-smoothed. By comparison, the proposed LWC-DenseFuse simultaneously preserves prominent infrared targets and rich visible-scene details. Pedestrian targets exhibit higher contrast and clearer boundaries, while fine-grained structures such as tree branches, roadside facilities, vehicle contours, and pavement textures are effectively retained. These results demonstrate that LWC-DenseFuse achieves a better trade-off between target enhancement and detail preservation, producing fused images with improved visual fidelity and scene representation.

The objective metrics of the comparative experiment with competing approaches on the MSRS and LLVIP datasets are presented in Table 4 and Table 5, respectively.

Table 4 and Table 5 quantitatively compare the competing methods in terms of fusion performance and computational efficiency. In terms of efficiency, the proposed LWC-DenseFuse exhibits a significant advantage. Compared with UNIFusion (0.39 M parameters), LWC-DenseFuse requires only 0.02 M parameters, corresponding to a reduction of approximately 95%. While LUT-Fuse reports the lowest FLOPs, LWC-DenseFuse achieves comparable computational complexity and retains the smallest parameter count among all compared methods. This lightweight design substantially reduces storage and deployment costs while still meeting the computational requirements of real-time applications.

In terms of fusion quality, despite its extremely lightweight architecture, LWC-DenseFuse achieves competitive and often superior performance. On the MSRS dataset, it attains the best EN and the second-best AG, indicating enhanced information richness and detail preservation. On the LLVIP dataset, it again achieves the highest EN and ranks second in both Qabf and AG. The strong performance in Qabf further demonstrates the effectiveness of the proposed LWC module in preserving edge and structural information from the source images.

Overall, the proposed LWC-DenseFuse achieves a favorable balance between model compactness and fusion performance. The results verify that the lightweight design and information compensation strategy effectively reduce feature redundancy while retaining critical complementary information, enabling high-quality fusion with minimal computational cost.

## 5. Discussion and Conclusions

This paper presented LWC-DenseFuse, a lightweight infrared–visible image fusion network built upon a self-compensating lightweight convolution framework. By explicitly compensating for the loss of feature interactions introduced by lightweight operations, the proposed method achieves an effective balance between fusion performance and computational efficiency. Building upon the self-compensating lightweight convolution module, this method achieves efficient model compression through depthwise separable convolution and linear transformation, while also reconstructing inter-channel information interaction by integrating strategies such as channel attention and channel shuffle. It successfully accomplishes the high-quality preservation of both infrared thermal radiation information and rich visible-light texture under an extremely low parameter count. The algorithm proposed in this paper has been validated on the TNO, MSRS, and LLVIP datasets. Through ablation and comparative experiments, the effectiveness of the proposed lightweight design and information compensation strategy is demonstrated. However, certain challenges remain when facing more complex real-world application scenarios, such as strong noise or smoke. Future work will focus on complex and extreme environments, aiming to further enhance the robustness and feature extraction capability of lightweight fusion networks in unstructured settings.

## Figures and Tables

**Table 1:** Comparison of Fusion Metrics for Different Methods on the TNO Dataset.

Model	TNO						
	AG (↑)	EN (↑)	VIF (↑)	Qabf (↑)	MI (↑)	Param/M (↓)	Avg_Time/s (↓)
M1	3.3433	6.4986	0.6949	0.3613	**2.2539**	**0.0194**	0.0169
M2	3.7295	6.5443	0.6694	0.3654	2.0457	0.0195	0.0110
M3	3.7295	6.0730	0.5088	0.2826	1.6519	0.0211	**0.0087**
M4	**4.7145**	**7.0764**	**0.7895**	**0.3945**	1.9464	0.0212	0.0112

Note: ↑ and ↓ indicate that higher and lower values are preferable, respectively. The best and second-best results are highlighted in bold and underlined, respectively.

**Table 2:** Overall Performance Comparison of Lightweight Convolution Methods on the MSRS.

Model	MSRS						
	AG (↑)	EN (↑)	VIF (↑)	Qabf (↑)	MI (↑)	Param/M (↓)	Avg_Time/s (↓)
DenseFuse	2.1084	5.9797	0.7199	0.4061	2.7603	0.0740	**0.0026**
DW-DenseFuse	2.2380	6.0436	0.7466	**0.4647**	**2.8583**	**0.0150**	0.0028
Ghsot-DenseFuse	4.7007	6.7492	0.6547	0.3509	2.1305	0.0550	0.0044
Star-DenseFuse	2.0641	5.9446	0.7007	0.3804	2.7000	0.1491	0.0080
LWC-DenseFuse	**5.1644**	**7.0520**	**0.8530**	0.3542	2.0939	0.0212	0.0106

Note: ↑ and ↓ indicate that higher and lower values are preferable, respectively. The best and second-best results are highlighted in bold and underlined, respectively.

**Table 3:** Overall Performance Comparison of Lightweight Convolution Methods on the LLVIP Dataset.

Model	LLVIP						
	AG (↑)	EN (↑)	VIF (↑)	Qabf (↑)	MI (↑)	Param/M (↓)	Avg_Time/s (↓)
DenseFuse	3.4885	6.8486	0.7405	0.4190	2.5696	0.0740	0.0028
DW-DenseFuse	3.6312	6.9010	0.7589	0.4652	2.6445	**0.0150**	**0.0023**
Ghsot-DenseFuse	**5.4603**	7.1307	0.7440	0.4750	2.1661	0.0550	0.0045
Star-DenseFuse	3.0758	6.8457	0.7280	0.3068	**2.7117**	0.1491	0.0084
LWC-DenseFuse	5.2757	**7.3922**	**0.8721**	**0.5446**	2.5154	0.0212	0.0092

Note: ↑ and ↓ indicate that higher and lower values are preferable, respectively. The best and second-best results are highlighted in bold and underlined, respectively.

**Table 4:** Comparison of Fusion Metrics with Different Methods on the MSRS Dataset.

Model	MSRS					
	AG (↑)	EN (↑)	Qabf (↑)	MI (↑)	Param/M (↓)	FLOPs/G (↓)
UNIFusion	4.7855	5.8130	0.4769	**3.6204**	0.3900	70.214
RCAFusion	**5.5645**	6.942	**0.6733**	2.6308	0.1823	59.382
SAGE	4.7569	6.900	0.4001	2.4063	0.1356	20.337
LUT-Fuse	4.9828	6.920	0.5650	2.7150	0.0730	**2.449**
ours	5.1644	**7.0520**	0.3542	2.0939	**0.0212**	8.842

Note: ↑ and ↓ indicate that higher and lower values are preferable, respectively. The best and second-best results are highlighted in bold and underlined, respectively.

**Table 5:** Comparison of Fusion Metrics with Different Methods on the LLVIP Dataset.

Model	LLVIP					
	AG (↑)	EN (↑)	Qabf (↑)	MI (↑)	Param/M (↓)	FLOPs/G (↓)
UNIFusion	**7.0098**	6.5163	0.4718	3.0997	0.3900	386.543
RCAFusion	3.8596	6.7008	**0.5851**	**4.6500**	0.1823	253.362
SAGE	3.1630	6.0038	0.4363	3.2402	0.1356	86.770
LUT-Fuse	3.8150	6.5649	0.4999	3.6483	0.0730	**10.449**
ours	5.2757	**7.3922**	0.5446	2.5154	**0.0212**	37.728

Note: ↑ and ↓ indicate that higher and lower values are preferable, respectively. The best and second-best results are highlighted in bold and underlined, respectively.

## Data Availability

The data is included in the article. We have established a cloud storage system, which sequentially stores the data of our ablation experiment, fusion result data, and test dataset. The online storage link is: https://pan.baidu.com/s/1D5NbVDKL9a3b1ZkMvGid3w?pwd=rcfw (accessed on 17 May 2026).

## Abbreviations

The following abbreviations are used in this manuscript:

AG	Average Gradient
CNN	Convolutional Neural Network
DW	Depthwise Separable Convolution
EN	Information Entropy
LWC	Lightweight Convolution
MI	Mutual Information
MSE	Mean Squared Error
SSIM	Structural Similarity
VIF	Visual Information Fidelity
